# An antibiotic cement-coated locking plate as a temporary fixation for treatment of infected bone defects: a new method of stabilization

**DOI:** 10.1186/s13018-020-1574-2

**Published:** 2020-02-11

**Authors:** Chao Jia, Xiaohua Wang, Shengpeng Yu, Hongri Wu, Jie Shen, Qiang Huang, Zhao Xie

**Affiliations:** 1 Department of Orthopedics, First Affiliated Hospital, Army Medical University (Third Military Medical University), Chongqing, 400038 People’s Republic of China; 2 Department of Orthopaedics, Traditional Chinese Medicine Hospital, Shaping Ba District, Chongqing, 400038 People’s Republic of China

**Keywords:** Bone infection, Infected bone defects, Internal fixation, Antibiotic cement-coated locking plate

## Abstract

**Background:**

The induced membrane technique has achieved good clinical results in the treatment of infected bone defects, and external fixation is the main method, but it causes inconvenience and complications in patients. In this study, our objective was to investigate the outcomes of using an antibiotic cement-coated locking plate as a temporary internal fixation in the first stage of the surgical induced membrane technique for treating extremities with infected bone defects.

**Methods:**

We retrospectively analysed patients with lower extremity infected bone defects in our department between January 2013 and December 2017. All patients were treated with the induced membrane technique. In the first stage, the defects were stabilized with an antibiotic cement-coated locking plate as a temporary fixation after debridement, and polymethyl methacrylate cement was implanted to induce the formation of a membrane. In the second stage, bone grafting rebuilt the bone defects after infection control, and the temporary fixation was changed to a stronger fixation.

**Results:**

A total of 183 patients were enrolled, with an average follow-up duration of 32.0 (12–66) months. There were 154 males and 29 females with an average age of 42.8 (10–68) years. The infection sites included 81 femurs, 100 tibias and 2 fibulas. After the first stage of treatment (infection control), 16 (8.7%) patients had recurrence of infection. In terms of the incidence of complications, 4 patients had poor wound healing, 2 patients had fixation failure and 1 patient had femoral fracture due to a fall. After the second stage of treatment (bone reconstruction), there were 24 (13.1%) recurrences of infection, with a mean time of 9.9 months (range 0.5 to 36). Among them, 18 patients underwent bone grafting after re-debridement, 6 received permanent placement of antibiotic bone cement after debridement and 2 patients refused further treatment and chose amputation. Bone healing was achieved in 175 (95.9%) patients at the last follow-up, and the average time to bone union was 5.4 (4–12) months.

**Conclusions:**

Antibiotic cement-coated locking plates have good clinical effects in the control of bone infection, but attention must be paid to the possible difficulty of skin coverage when applied in calves.

## Introduction

Bone infection is a major problem in the department of orthopaedics. Sufficient stabilization and thorough debridement are conducive to the treatment of infection. The external fixator is a widely accepted form of fixation that provides stability in the treatment of bone infections [1], which can not only provide stability but also prevent the formation of bacterial biofilms, which always increase the recurrence of infection [2]. However, external fixation has many complications, such as pin track infection [3], poor stability, joint stiffness and adverse effects on daily life. To avoid these deficiencies and shortcomings, some scholars have attempted to use internal fixations in bone infection. Liporace [4] and Conway [5] attempted to treat infected bone fractures with antibiotic cement-coated locking plates. However, the study had few cases and short follow-up times, and further analysis of the long-term efficacy and application conditions or complications is incomplete. In this study, we report a series of patients with extremity infections treated with antibiotic cement-coated locking plates as internal fixation in the first stage of the induced membrane technique, followed by grafting to rebuild bone defects after formation of the induced membrane. Our aim was to investigate the outcomes of this new method and to provide a reference for clinicians when applying this method.

## Patients and methods

This study was approved by the ethics committee of our hospital, and informed consent was obtained from the patients for the publication of individual clinical details and accompanying images. We retrospectively analysed patients with lower extremity infected bone defects in our department between January 2013 and December 2017. Diagnosis of bone infection was made based on local bone pain, rubor and localized swelling, or a draining sinus tract during examination, imaging procedures, microbiological and histopathological examinations and laboratory studies [6]. Our inclusion criteria were as follows: (1) lower extremity bone infection patients admitted to our department; (2) treatment with the induced membrane two-stage technique, with antibiotic cement-coated locking plates used as temporary internal fixation after debridement; (3) bone defects remaining after debridement; and (4) follow-up time > 12 months. The exclusion criteria were as follows: (1) malignant tumours; (2) serious vascular and nerve injuries with no possibility of limb salvage; and (3) incomplete follow-up data.

### Surgical technique

In the first stage, thorough debridement was performed, and a locking plate coated with antibiotic cement was used to stabilize the bone defects. The bone defects were filled with antibiotic bone cement to induce the formation of the induced membrane. In the second stage, bone grafting was performed to rebuild bone defects.

#### Stage I (infection control)

X-ray, MRI and radionuclide examination were used to determine the approximate infectious area. The sinus, sequestrum, necrotic tissue and ischaemic sclerotic bone were thoroughly eradicated. Aggressive debridement utilising a high-speed saline-cooled burr removed necrotic bone, granulation tissue was eliminated with a rongeur, and the membrane curetted off the bed of bone. All infected and nonviable bone and soft tissue was cleared until good punctate haemorrhage appeared (characterized by a “paprika sign”). Specimens of infected bone and infected tissue were sent for pathological examination and microbiological examination. The wound was irrigated with a large amount of iodophor, hydrogen peroxide and saline. A locking compression plate was used to stabilize the femur, and a reconstruction locking plate was used to stabilize the tibia and fibula. Antibiotic cement (0.5 g of gentamicin per 40 g of PMMA) (Heraeus, Hanau, Germany) mixed with 5 g of vancomycin was used to fill the bone defects. The plate was wrapped and extended 1–2 cm to the proximal and distal ends to expand the volume of the induced membrane. Free flaps or local transfer flaps were used for some patients to ensure full wound coverage. Drainage tubes were placed before suturing. Effective intravenous antibiotics were administered after operation based on the sensitivity of the isolated bacteria, and a third-generation cephalosporin (ceftazidime) was administered if no bacteria were isolated. Patients were encouraged to exercise in bed. If infection recurred, the first-stage operation was performed repeatedly.

#### Stage II (bone reconstruction)

At least 6 weeks after the first stage of treatment, when the soft tissue condition was good, there were no clinical signs of infection (swelling, fever, pain, sinus formation and so on); blood tests (white blood cells (WBCs), C-reactive protein (CRP), and erythrocyte sedimentation rate (ESR)) returned to normal; and the imaging examination did not indicate infection, so the second stage of treatment was feasible. The former incision was chosen, and the induced membrane was cut longitudinally to carefully protect the integrity of the induced membrane. The bone cement and internal fixation were removed thoroughly, and the bone ends were decorticated. Autografts were harvested from the iliac crest (monolateral or bilateral), and allografts were mixed for patients with large bone defects. An intramedullary nail, a locking plate or an intramedullary nail attached to a locking plate was selected for final stabilization. The induced membrane was sutured carefully, and then the incision was sutured completely.

### Postoperative follow-up

Follow-up was performed every month after the first stage and every 3 to 6 months after the second stage. The review included clinical symptoms and signs of infection (such as swelling, fever, pain and sinus formation), laboratory examinations (WBC, ESR, CRP) and anteroposterior and lateral X-ray examination. The recurrence of infection, bone healing and complications were observed and recorded. When the WBC, ESR and CRP returned to normal and there were no clinical signs of local infection (such as swelling, fever, pain and sinus formation), no recurrence of infection was considered to have occurred. The bone union time was obtained by X-ray, and radiological union was defined as three-sided cortical bridging on two perpendicular X-rays of the defect zone [7].

### Statistics

Data analysis was performed using SPSS 23.0 statistical software (SPSS Inc., USA). The measurement data is represented with ranges, and the enumeration data are expressed by rate. Enumerated data were compared using Pearson or Fisher’s exact tests. The difference between the groups was considered to be significant when *p* < 0.05 in a two-sided test.

## Results

A total of 183 patients with bone infection were enrolled. There were 154 males and 29 females with an average age of 42.8 (10–68) years. The mean follow-up time after bone reconstruction was 32.0 (12–66) months. The infection sites included 81 femurs, 100 tibias and 2 fibulas. According to the aetiological classification proposed by Waldvogel [8], there were 35 cases of haematogenous osteomyelitis, 148 cases of post-traumatic osteomyelitis of which 54 had secondary to internal fixation of closed fractures and the other 94 had secondary to open fractures. According to the Cierny-Mader anatomic classification [1], there were 48 patients with type III and 135 patients with type VI. According to the Cierny-Mader physiologic classification [1], there were 109 patients of class A and 85 patients of class B. The duration of bone infection before admission ranged from 0.5 to 540 months. The average length of bone defected was 7.7 (1.5–22.7) cm. Among the 183 patients, 113 (61.7%) had positive bacterial isolations. Among those 113 patients, 92 had cases of single bacterial infections, and 21 had cases of mixed bacterial infections. Of these, 56 (49.6%) were infected with *Staphylococcus aureus* (including 16 with methicillin-resistant *Staphylococcus aureus* (MRSA), 13 with *Enterobacter cloacae*, 11 with *Pseudomonas aeruginosa* and 9 with *Escherichia coli*. However, 69 patients had negative bacterial cultures (data shown in Table 1).

**Table 1:** Patients’ data from the 183 patients

Number (male-to-female), *n*		183 (154:29)
Mean age (range), years		42.8 (10–68)
Site (femur; tibia; fibula), *n*		81; 100; 2
Duration of bone infection (range), months		0.5–540
Aetiology (no. of patients)	Post-traumatic	148
	Closed fractures	54
	Open fractures	94
	Haematogenous	35
Organisms (no. of patients)	*Staphylococcus aureus (MRSA)*	56 (16)
	*Enterobacter cloacae*	13
	*Pseudomonas aeruginosa*	11
	*Escherichia coli*	9
	Negative	69
Cierny-Mader classification	III	48
(no. of patients)	IV	135
Physiologic class	A	103
	B	80
Smoker		70
Follow-up (12–66 months)	Extra revision Infection-free bone healing	26/183 175/183

After the first stage of treatment for infection control, 16 (8.7%) patients had recurrence of infection; 12 patients underwent two debridements, and 4 patients underwent three debridements before the infection was controlled. Complications occurred in 7 (3.8%) patients, including delayed union of the incision in 4 patients with a tibia infection; complications related to internal fixation in 3 patients: a femoral fracture resulting from an accidental fall in 1 patient and screw loosening in 2 patients with a long segmental bone defect of the femur. There was a significant difference in the recurrence rate between type A (4/103) and type B (12/68) patients after the first stage of treatment (*p* = 0.008 < 0.05). There was no significant difference in the recurrence rate among different sites of bone infection (*p* = 0.220 > 0.05) (data shown in Table 2).

**Table 2:** Possible predictors of revision after first-stage treatment

Group (*n*)	Recurrent revision (*n*)	Infection controlled (*n*)	*p* value
Total (183)	16	167	
Gender			
Male (154)	15	139	0.458
Female (29)	1	28	
Age			
< 40 years (88)	7	81	0.716
≧ 40 years (95)	9	86	
Physiologic class			
Type A (103)	4	99	0.008
Type B(80)	12	68	
Aetiology			
Post-traumatic (148)	12	136	0.770
Haematogenous (35)	4	31	
Classification			
Type III (48)	4	44	1.000
Type IV (135)	12	123	
Location			
Femur (81)	7	74	0.220
Tibia (100)	8	92	
Fibula (2)	1	1	
Number of previous operations			
≥ 1 (165)	13	152	0.519
< 1 (18)	3	16	
Smoker			
Yes (70)	9	61	0.121
No (113)	7	106	

**Fig. 1:**
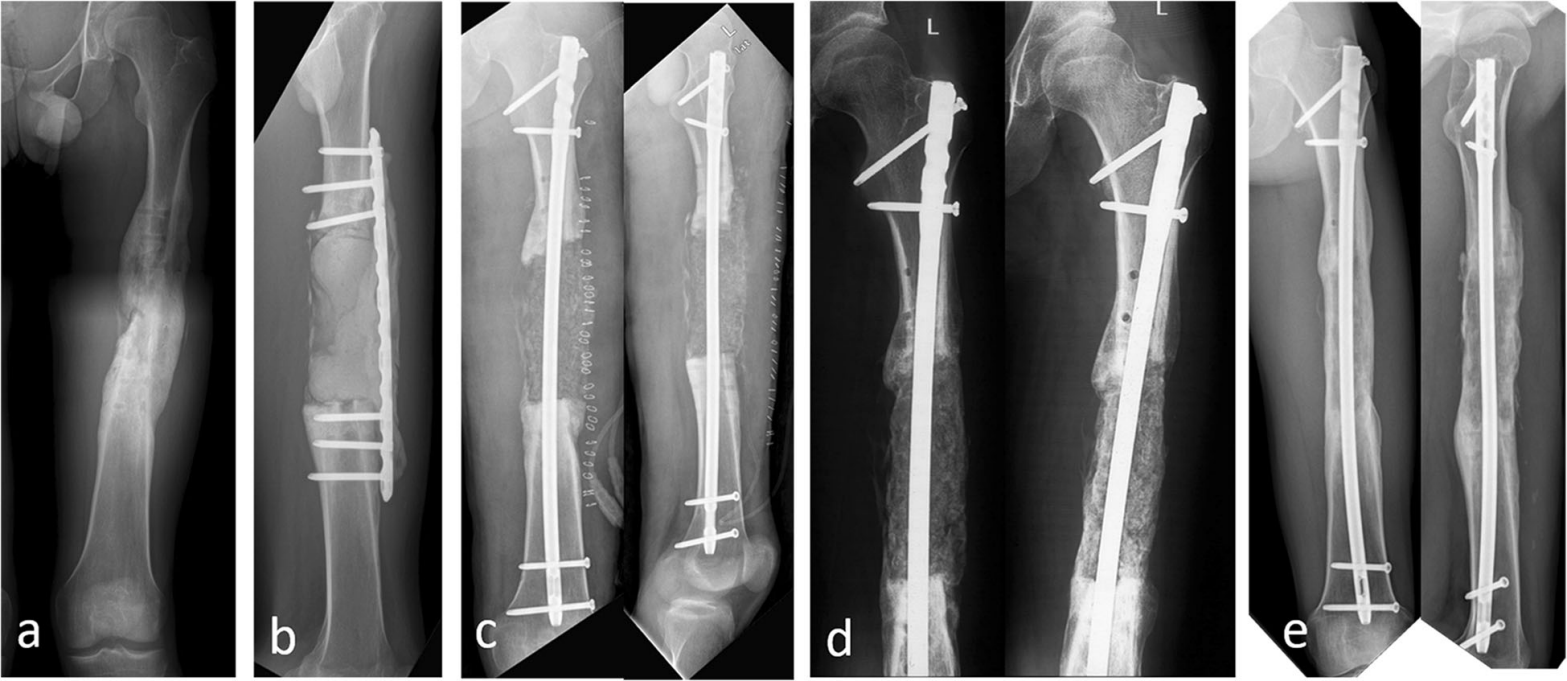
Twenty-one-year-old male suffered from bone infection of the left femur for 2 years. The result of bacterial culture during operation was *Enterobacter cloacae*. **a** X-ray shows bone destruction. **b** Resection of the lesion; antibiotic cement filled the defect and wrapped the plate. **c** Bone graft after 8 weeks. **d** Callus growth was observed after 3 months. **e** Bone union after 24 months

**Fig. 2:**
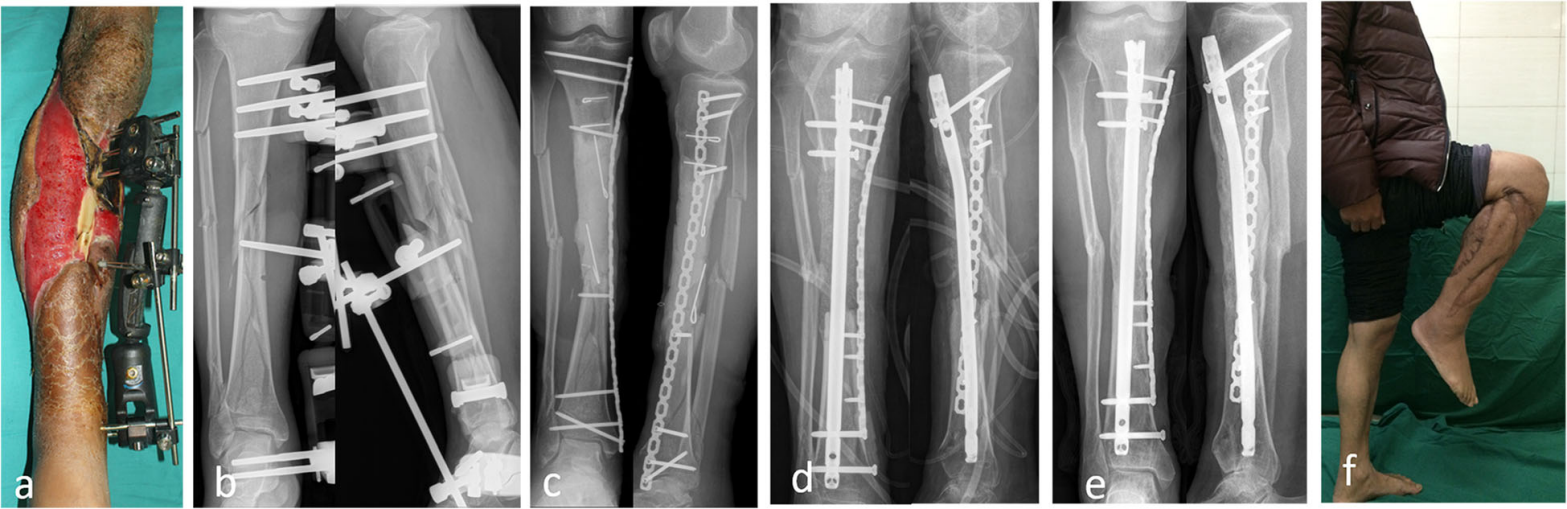
Forty-seven-year-old male. Bone infection after open injury. The culture results showed *Pseudomonas aeruginosa*. **a** Extensive soft tissue defect and bone exposure. **b** Preoperative X-ray. **c** Resection of the lesion. Antibiotic cement filled the defect and wrapped the plate. **d** Bone graft after 4 months. **e** Bone union after 24 months. **f** Function well

**Fig. 3:**
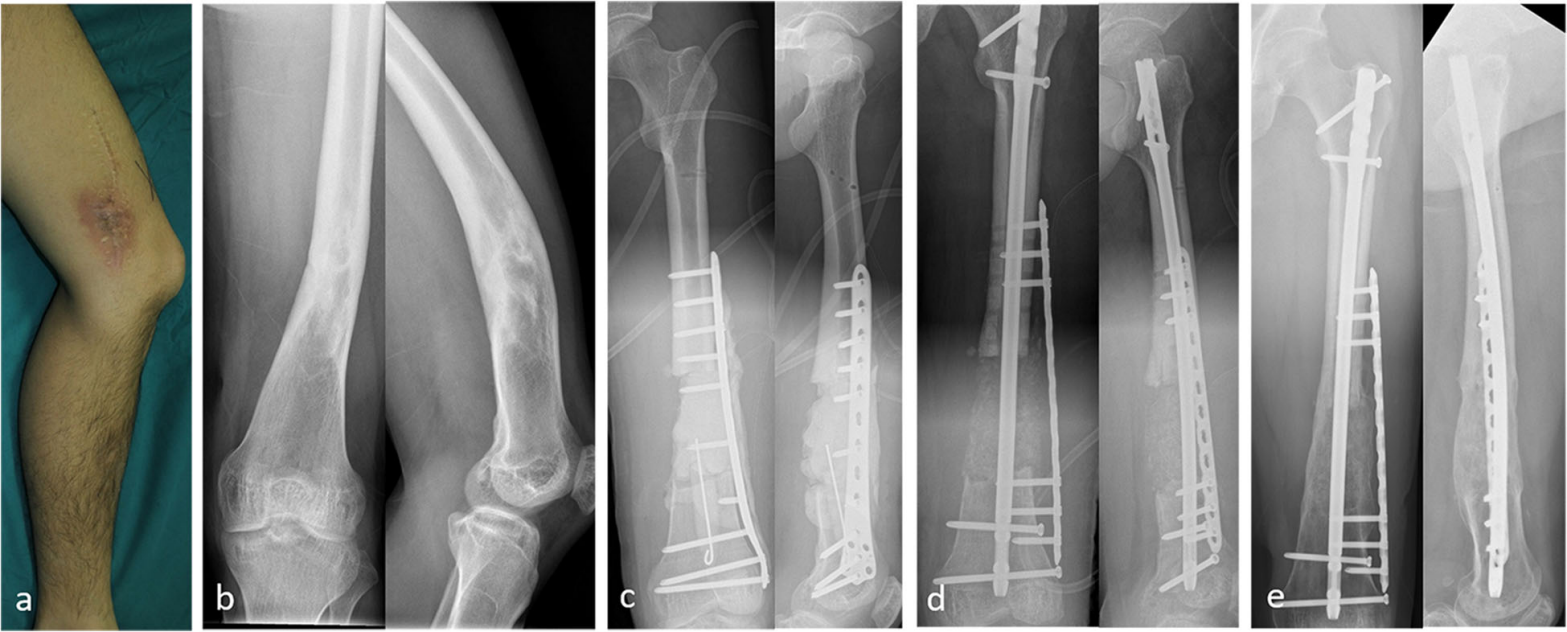
Thirty-year-old male. Bone infection of the right femur for 18 years. The culture results showed *Staphylococcus aureus*. **a P**reoperative photo. **b** Preoperative X-rays. **c** X-rays after the first stage. **d** X-rays after the second stage. **e** Bone union after 24 months

**Fig. 4:**
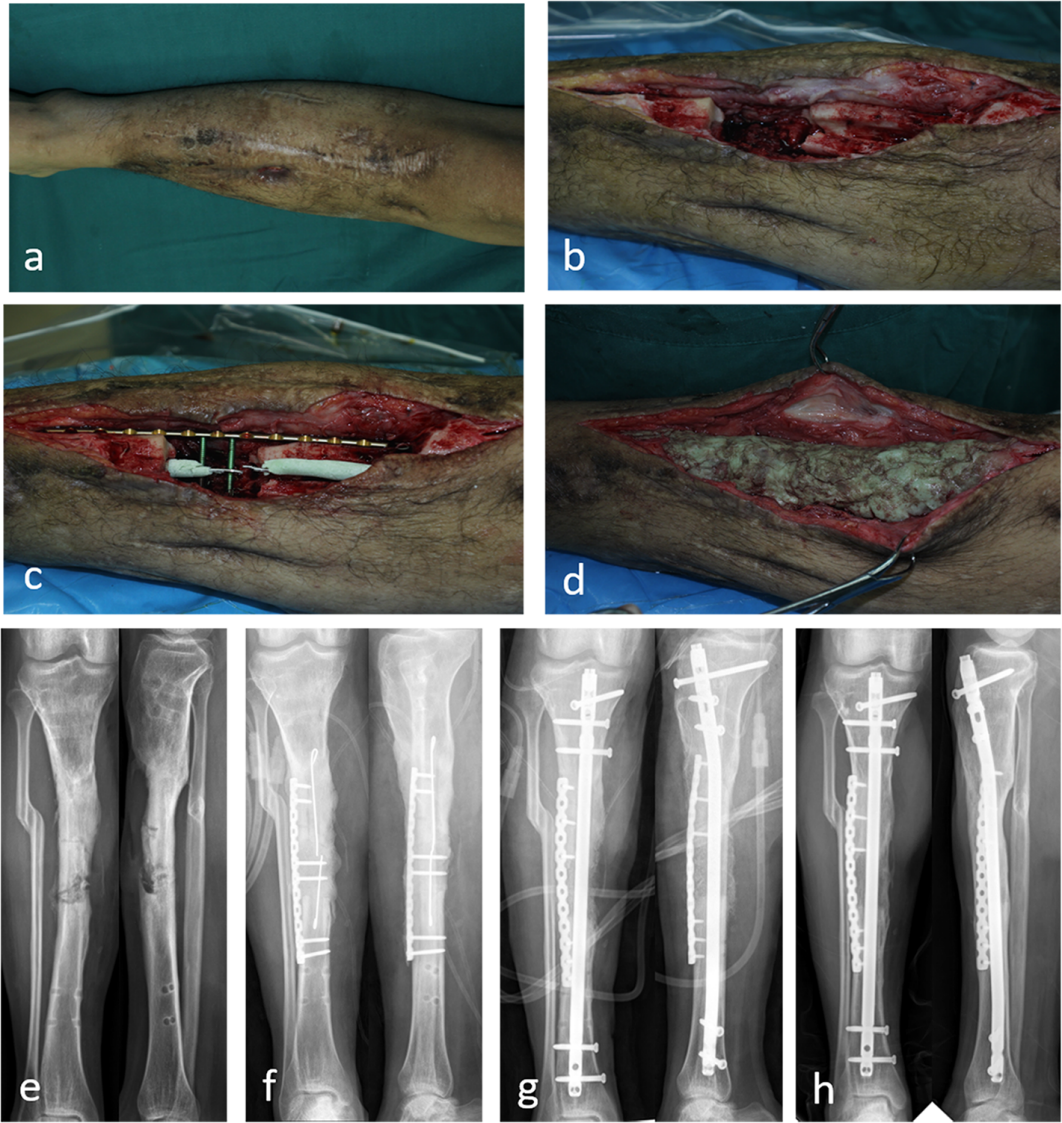
Twenty-three-year-old male. Bone infection after open reduction and internal fixation for 3 years. Deep tissue culture revealed *Staphylococcus aureus*. **a** Preoperative photo. **b** After debridement. **c** Reconstructive locked plate fixation. **d** Antibiotic bone cement filled with the defects and wrapped the fixation. **e** Preoperative X-rays. **f** X-rays after the first stage. **g** X-rays after the second stage. **h** Bone union after 36 months

After the second stage of treatment for bone reconstruction, there was recurrence of infection in 24 (13.1%) patients. Bone reconstruction was not performed in 6 elderly patients, and permanent antibiotic bone cement was implanted after radical debridement. Two patients refused further treatment and opted for amputation. Eighteen patients repeatedly underwent debridement and bone grafting. Finally, 175 patients (95.6%) achieved radiographic bone union within an average of 5.4 (4–12) months. (Typical cases are presented in Figs. 1, 2, 3 and 4).

## Discussion

Bone infection is one of the most challenging conditions in the field of orthopaedics. Its essence is a bacterial biofilm [9], which can resist host immunity and antibiotics [10, 11]. Bacteria easily attach to the surface of the metal surface to form biofilms, leading to infection and relapse [12, 13]. Therefore, internal fixation is not recommended in the presence of bone infections. As a widely accepted instrument, an external fixation can span the infected area and avoid the exposure to metal foreign bodies [14]. However, pin tract infection is the major complication of external fixation, which can decrease the stability [3]. When the infected site is close to the joint, trans-articular fixation leads to joint stiffness [15], which is not conducive to the recovery of joint function. External fixation is bulky, which affects patients’ ordinary life [16]. Long-term placement of the external fixation also has a negative impact on patients’ mental health [17]. Mark [18] reported 11 cases of femur infection treated with plate internal fixation after complete debridement. Alemdar [19] reported that 15 cases of deep infection after lower extremity fracture were treated with “non-contact” plate internal fixation on the basis of complete debridement, but no matter how thorough the debridement was, bacteria still existed [20], and residual bacteria could form biofilms on the surface of the internal fixation, resulting in recurrence of infection.

Wrapping with antibiotic bone cement can prevent bacteria from adhering to the surface of the internal fixation, and the high concentrations of antibiotics released from antibiotic bone cement can kill the residual bacteria after debridement, preventing the internal fixation from becoming a new source of infection [21, 22]. Our research applied internal fixations with locking plates coated with antibiotic bone cement for the treatment of 183 patients with lower extremity infection. The infection control rate was 91.3% after the first debridement. It has been proven from clinical practice that internal fixation can be used after infection debridement, which breaks through the prohibition of internal fixation in the case of bone infection. This method did not seem to cause more infectious complications compared to external fixation, but more importantly, patients were comfortable and complications associated with external fixation were avoided. Thorough removal of infected bone and soft tissue is the prerequisite for the use of internal fixation, and it is also the core for bone infection treatment [23, 24]. The goal of debridement is to achieve a viable vascularized environment and restore local immunity. The local high concentrations of antibiotics also play a key role. Antibiotics added to bone cement are slowly released over time. A high dose of antibiotics surrounds the plate to achieve a minimum local inhibitory concentration. These measures can prevent biofilm formation and thus reduce the infection recurrence rate [22, 25–27]. Furthermore, good stability is beneficial to the treatment of infection [28, 29]. It was confirmed in animal experiments that the infection rate in the unstable group was more than twice as high as that in the stable group [30]. The locking plate provides angular stability and axial stability [31], which provides excellent anchoring in osteoporotic bone. It can also help protect joint function by avoiding trans-articular fixation when bone infection occurs in the metaphysis. In addition, the locking plate does not contact the bone cortex and helps to protect the periosteal blood supply.

In this study, all patients were treated with antibiotic cement-coated locking plates. After the first stage of treatment, 16 (8.7%) patients experienced recurrence of infection, which manifested as wound nonunion, sinus formation, local redness, swelling and pain. After repeated debridement and internal fixation, the infection was controlled, and bone grafting progressed smoothly. We found that the recurrence rate of type B (17.6%) patients after debridement was significantly higher than that of type A patients (3.9%) (*p* < 0.05). This finding is consistent with Simpson’s prospective study. Simpson [32] found that patients defined as type B had a higher recurrence rate than patients defined as type A and that type B patients were recommended for more extensive debridement.

Delayed local skin healing occurred in the tibia of 4 patients. Due to long-term infection and repeated operations, the local soft tissue condition was poor, and the soft tissue defect after debridement made the incision suture tension larger. Therefore, skin flaps are recommended to ensure tension-free suturing of the incision. Reconstruction of the soft tissue envelope and establishment of a good blood supply can enhance local anti-infection capacity. Systemic antibiotics are more likely to be effective locally. Limb elevation and anti-swelling drugs were used after the operation. Fracture or loosening of the internal fixation is related mainly to weight bearing and accidental falls. In the first stage of treatment, the goal of the locking plate is to provide enough stability to allow patients to have a comfortable outpatient experience while controlling infection and to perform functional exercises. Considering that long plates will increase the difficulty of infection treatment, we chose a shorter plate, which may not be strong enough. Therefore, patients are recommended to walk with crutches without weight-bearing or partial weight-bearing after the first stage.

The induced membrane technique [33] proposed by Masquelet in 2000 is a new method for the treatment of bone defects. The membrane prevents the absorption of the graft bone and produces factors that promote osteogenesis [34], and the bone union rate is 80 ~ 100 % [35]. In this study, 175 (95.6%) patients obtained radiological bone union, and the union time was 5.4 months (range 4 to 12 months). When using locking plates for internal fixation, the plate should be completely wrapped in bone cement to ensure the integrity of the induced membrane and avoid possible residual bacterial adhesion. Furthermore, the coverage is extended to the bone tip to increase the volume of the induced membrane. Antibiotic-impregnated cement can not only release a high concentration of antibiotics but also induce a highly vascularized membrane rich in various bone factors [36], which provides a good environment for bone reconstruction.

Although this study has a good success rate and breaks through many traditional theoretical and technical taboos, there are still some deficiencies. First, this study is a retrospective study, and it can only be summarized from clinical experience for some postoperative complications but not confirmed from the experiment. Second, there is no control group and no comparison with other established methods.

## Conclusion

Temporary internal fixation with an antibiotic cement-coated locking plate has good clinical effect in the control of long bone infection in the lower extremities and is practical in all parts of the lower extremities, but the application premise must be thoroughly debrided. At the same time, poor soft tissue condition is a relative concern. Attention should be paid to the skin coverage of the lower leg.

## Data Availability

The datasets used and analysed during the current study are available from the corresponding author on reasonable request.

## Abbreviations

CRP: C-reactive protein
ESR: Erythrocyte sedimentation rate
MRI: Magnetic resonance imaging
MRSA: Methicillin-resistant *Staphylococcus aureus*
PMMA: Polymethyl methacrylate
